# Uptake of Peritoneal Dialysis by Minoritized Patients: The Role of Modality Education

**DOI:** 10.34067/KID.0000000702

**Published:** 2025-02-03

**Authors:** Ivelina Arnaoudova, Clara Wilson, Katherine Rizzolo, Jenny Shen, Nahian Ehtesham, Jennifer Wilson, Ladan Golestaneh

**Affiliations:** 1 Division of Nephrology, Montefiore Medical Center, Bronx, New York; 2 Section of Nephrology, Boston University Chobanian and Avedisian School of Medicine and Boston Medical Center, Boston, Massachusetts; 3 Division of Nephrology, The Lundquist Institute at Harbor-UCLA Medical Center, Torrance, California; 4 Williamsbridge Home Dialysis Facility, DaVita Kidney Care, Denver, Colorado; 5 Section of Nephrology, Yale University School of Medicine, New Haven, Connecticut

**Keywords:** CKD, nephrology, peritoneal dialysis

## Abstract

**Key Points:**

- In this study of minoritized patients referred to modality education before dialysis, a high proportion selected, but fewer initiated dialysis with, peritoneal dialysis (PD).
- Those who initiated with PD were younger, healthier, and more responsive to education staff than those who started with hemodialysis.
- Patients identified lack of emotional support and information about overcoming structural barriers to PD as shortfalls in the education program.

**Background:**

In a cohort of patients with late-stage kidney disease who completed dialysis modality education and who self-identified as racial ethnic minorities, we studied characteristics of those choosing peritoneal dialysis (PD) and perception of usefulness of the education session in modality selection.

**Methods:**

In this study of individuals with kidney failure cared for by nephrologists at Montefiore Medical Center, Bronx, New York, who were referred for modality education, we (*1*) tested the association of patient characteristics with modality selection in 113 patients from 2021 to 2023 and (*2*) examined patient perception of the quality of modality education from 13 semistructured interviews. We compared sociodemographic, clinical attributes, and patient responsiveness to attempts made by staff among those who selected and initiated PD with those who (*1*) did not select PD or (*2*) initiated on hemodialysis urgently. We performed qualitative analysis of interviews to reach consensus on theoretical domain framework concepts and how they fit events in the kidney failure trajectory.

**Results:**

Compared with individuals who required urgent hemodialysis, those who selected and were initiated on PD were younger (54 versus 66 years), had fewer comorbidities, and did not require as many attempts to schedule modality education. Qualitative analysis of interviews showed that experience with staff and quality of information conveyed during education was generally positive, but the following gaps were identified: lack of support for the emotional trauma of kidney failure diagnosis, inability to address structural barriers to PD specific to the patient population, and the lack of a deliberate program to lessen anxiety about the responsibility of PD.

**Conclusions:**

Incorporation of tailored content that addresses clinical comorbidity, structural barriers to care, and emotional trauma constitute aspects of modality education that can be improved to increase PD uptake among minoritized patients.

## Visual Abstract

**Figure:**
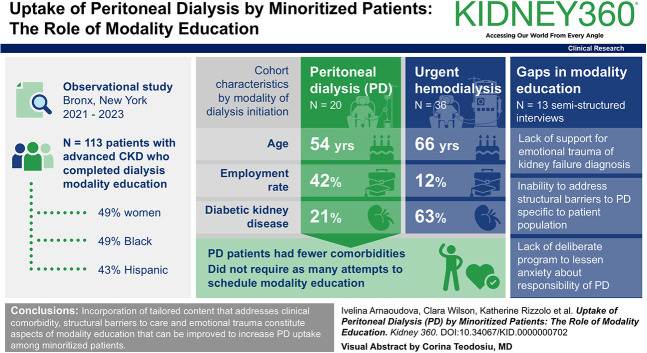

## Introduction

Peritoneal dialysis (PD) shows equivalent clinical outcomes to in-center hemodialysis and offers additional patient-centered benefits such as enhanced quality of life and higher levels of patient autonomy.^1^ While policymakers and payers have incentivized uptake of home dialysis modalities such as PD through novel payment structures, home dialysis therapies remain underutilized in the United States, especially among minoritized groups who have high rates of kidney failure. In 2022, 14% of White patients initiated dialysis with PD compared with 10.4% of Black and 11.8% of Hispanic incident dialysis patients.^2^ These trends are not completely explained by medical, demographic, or socioeconomic factors historically associated with lower access to home dialysis.^3^ A recent study of United States Renal Data System data by Shukla *et al.* demonstrated that predialysis kidney disease education, which is disappointingly provided at a low rate to Black and Hispanic patients, acts as a mediator in uptake of home dialysis.^4^ Minoritized populations, who are less likely to receive dialysis modality education or predialysis care, report not having had the full picture regarding kidney disease and treatment options at the start of dialysis and more commonly have negative feelings and distrust compared with their White counterparts.^5^ The transition to dialysis, especially if urgent, is accompanied by emotional trauma, which may also hinder patient engagement in modality decision-making.^6^ The awareness of this trauma is frequently missed by clinician educators,^7,8^ which decreases patients' trust, while traditional dialysis modality educational programs seldom acknowledge it as a barrier to patient-centered decision-making.^8–10^

The paucity of culturally concordant modality education, with tailored emotional support, contributes to the low uptake of home modalities among minoritized individuals. For example, Latinx populations with kidney failure report a lack of timely dialysis modality education, as well as home dialysis stigma within their communities, leading to fear of home dialysis therapies.^11^ Most are more likely to use their social networks, especially peer to peer information, when making important health care decisions, while some also describe using religion and social networks for health care decision-making support,^12^ a resource also used by undocumented Latinx populations.^13^

The aim of this study was to (*1*) explore what practical and timing characteristics of modality education are associated with PD uptake and (*2*) to understand the perceptions of formal predialysis modality education and its influence on shared decision-making and home dialysis uptake in a cohort of Black and Hispanic individuals with kidney failure.

## Methods

### Identification of Correlates of Home Dialysis Initiation

#### Study Design and Population

In this observational study, we analyzed prospectively collected data on a cohort of 113 adult individuals who were referred by their nephrologists at Montefiore Medical Center to, and attended, in-person dialysis modality education from April 2021 to October 2023. We identified correlates of PD initiation. The education session was offered in-person by a large dialysis organization at a local dialysis facility before dialysis initiation. At the time of referral, the referring nephrologist informed the patient that a member of the research team may reach out to him/her about participation in the qualitative portion of this study.

#### Data Source and Variables of Interest

Data sources included modality education staff notes and Montefiore Medical Center electronic health records available for chart review. Patients were followed for an average of 12 months. Potential correlates of PD update included (*1*) sociodemographic characteristics of patients, (*2*) the timing of receipt of education (number of days between referral and attended session), (*3*) ease of modality education referral and scheduling (number of contact attempts by educational team before scheduling), and (*4*) frequency of post modality education contact (Table 1).

**Table 1:** Comparison of those who ultimately elected PD compared with those who ultimately elected hemodialysis and those who were undecided

Variable	Ultimately Selected PD (*n*=49)	Ultimately Selected Hemodialysis (*n*=36)	Undecided (*N*=28)	*P* Value
Age, median (IQR)	63.0 (51–68)	66.5 (54–75)	68.5 (59–75.5)	0.05
**Sex, *n* (%)**				1.0
Female	24 (49.0)	18 (50)	13 (46.4)	
**Race, *n* (%)**				0.1
Asian	14 (28.6)	19 (52.8)	6 (21.4)	
Black	24 (49.0)	13 (36.1)	18 (64.3)	
Other	3 (6.1)	1 (2.8)	1 (3.6)	
White	8 (16.3)	3 (8.3)	3 (10.7)	
**Ethnicity, *n* (%)**				0.02
Hispanic	19 (38.8)	22 (44.9)	8 (16.3)	
Married or significant other, *n* (%)	22 (44.9)	15 (41.7)	10 (35.7)	0.7
**Insurance, *n* (%)**				0.2
Yes	49 (100)	34 (94.4)	27 (96.4)	
**Employment status, *n* (%)**				0.3
Unemployed	9 (18.4)	8 (22.9)	4 (14.3)	
Employed	16 (32.6)	7 (20.0)	6 (21.4)	
Retired/disability	24 (49.0)	18 (51.4)	15 (53.6)	
Other	0	2 (5.7)	3 (10.7)	
**Cause of ESKD, *n* (%)**				0.3
DM	25 (51.0)	23 (63.9)	12 (42.9)	
HTN	9 (18.4)	6 (16.7)	6 (21.4)	
GN or PCKD	15 (30.6)	6 (16.7)	8 (28.6)	
Other	0	1 (2.8)	2 (7.1)	
**Charlson comorbidity score category, *n* (%)**				0.2
Low (2–5)	24 (49.0)	13 (36.1)	9 (34.6)	
Medium (6–7)	19 (38.8)	12 (33.3)	9 (34.6)	
High (8–15)	6 (12.2)	11 (30.6)	8 (30.8)	
eGFR at initial nephrology visit, median (IQR)	19 (9–26)	23.5 (16–31)	17 (9.5–23.5)	0.03
eGFR at modality referral, median (IQR)	13 (10–17)	12.5 (10.5–15)	12.5 (10–16)	0.6
Median number of days between referral and education, *N* (IQR)	26 (8–43)	21 (11.5–43)	21.5 (1–37.5)	0.3
No. of attempts made to schedule education, *N* (IQR)	1 (1–3)	2 (1–5)	3 (2–4.5)	0.004
Median number of nephrology appts scheduled in year before referral, *N* (IQR)	5 (3–7)	5 (4–7)	4 (2–6)	0.3
Median number of missed nephrology appts in year before referral, *N* (IQR)	1 (0–2)	1 (0–2)	1 (0–2)	0.7
Nephrologist educated patient before referral, yes	42 (85.7)	32 (88.9)	18 (64.3)	0.02
Median number of contacts made by education team within 1 yr after education, *N* (IQR)	5 (3–7)	3 (2.5–6.5)	3 (2.5–6.4)	0.4

Appts, appointments; DM, diabetes mellitus; HTN, hypertension; IQR, interquartile range; PCKD, polycystic kidney disease; PD, peritoneal dialysis.

#### Outcome

Our primary outcome was selection of PD, and initiation of dialysis with PD, after education. An initial dialysis selection was made at the time of modality education; undecided patients were marked as such. An ultimate modality selection was made during a nephrology visit after the modality education. We also captured the actual first modality used by patients who initiated dialysis during the time frame of the study.

#### Statistical Analysis

We used descriptive statistics as appropriate to report patient characteristics. We compared sociodemographic and clinical attributes of patients who ultimately selected PD with those who ultimately selected hemodialysis using chi-square and Wilcoxon rank sum test. Of those who initiated dialysis during follow up (*n*=60), we compared attributes of patients who selected and initiated PD with those who required urgent hemodialysis, regardless of initial or ultimate modality choice. In this way, we hoped to illustrate patient and referral attributes associated with PD initiation as compared with a nonoptimal urgent hemodialysis initiation.

### Semistructured Interviews of Patients and Their Experience with Predialysis Education

#### Study Design and Participants

We completed 13 semistructured interviews of adult patients with advanced CKD (stages 4, 5, and ESKD) who received modality education. Patients were selected using a convenience sample from a database of patients referred for education, which was pulled using the electronic health record. Eligibility included cognitive ability and willingness to engage in an interview about their experiences. While all patients were pulled from one database, some patients were approached by their nephrologists about the study, while others were cold called by the interviewing researcher.

#### Data Collection

One researcher with 5 years of in-depth interview experience conducted all 13 interviews to ensure consistent questioning across patients (I. Arnaoudova). She had no relationship with the patients, minimizing bias. Interviews were conducted by phone call through Doximity or Microsoft teams, and all patients verbally consented at the beginning of the call. We were interested in patients' perceptions of the modality education session and how this influenced their modality decisions. We selected several *a priori* domains on the basis of the theoretical domain framework (TDF) for the interview guide and analysis (see Supplemental Material for the interview guide).^14^ TDF was chosen because it addresses important theoretical categories that enable identification of determinants of behavior and decision-making. The 13 interviews were conducted between February 2023 and July 2023. Interviews were audio-recorded, transcribed verbatim, deidentified, and continued until thematic saturation was achieved. Participants received a $25 gift card. We followed the Consolidated Criteria for Reporting Qualitative Research reporting guideline.^15^ The Einstein/Montefiore Institutional Review Board (IRB) approved this study (IRB: 2022-14543). This study followed the Declaration of Helsinki.

#### Analytical Methods

Interview transcripts were analyzed independently by three study members using Excel and Word between March 15, 2024, and June 18, 2024 (N. Ehtesham, I. Arnaoudova, and C. Wilson). The TDF framework was used to deductively identify convergent themes, then participant responses were inductively analyzed to generate overarching themes. These concepts were grouped into initial themes and subthemes using the TDF framework and principles of grounded theory. Coders reached consensus on themes and subthemes after review of the findings to ensure that these findings reflected the full range and depth of the data. Member checking consisted of the study team leading a series of virtual meetings and repeating consensus reached on domains, themes, and content of themes after analysis of each transcript.

## Results

### Identification of Correlates of Home Dialysis Initiation

#### Population Selection

**Figure 1:**
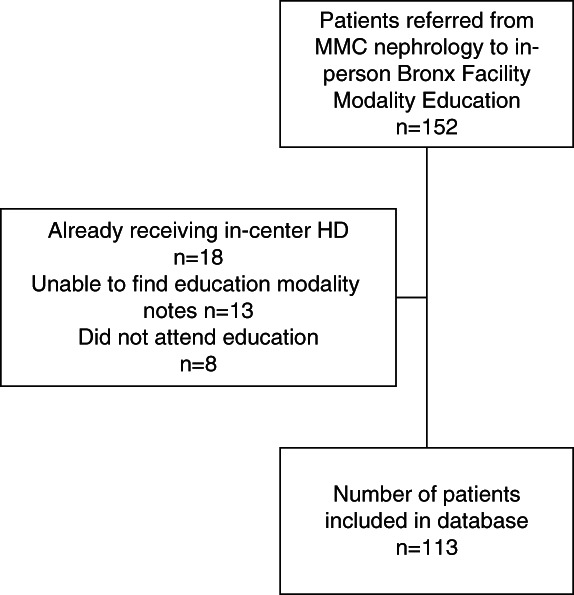
**Consort diagram for prospective cohort of patients referred for modality education.** HD, hemodialysis; MMC, montefiore medical center.

Of the 152 patients who had been referred for modality education, we included 113 after excluding those who had already received in-center hemodialysis, those without documentation of completion of education, and those who did not attend class (Figure 1).

#### Patient Characteristics

The median age was 66 years (interquartile range [IQR], 54–72). Of the 113 patients, 49% were women, 49% were Black, and 43% self-identified as Hispanic. The median Charlson score was 6 (IQR, 5–7), and the most common cause of progressive CKD was diabetes (53%). Regarding socioeconomic factors, 42% were married or had a significant other, 97% had some form of insurance, and 77% were employed or receiving disability. The mean eGFR at initial nephrology referral was 20.4 cc/ml per minute (SD, 11.1), and at referral for modality education, it was 13.6 cc/ml per minute (SD, 5.2). Of the 113 patients referred for modality education, 21 (18%) did not receive prior education from their nephrologists. The median number of attempts made by staff to schedule education was 2 (IQR, 1–4), and the median number of days between referral and completed education was 23 (IQR, 7–41). The median number of contacts by the education team within 1 year after education was 3 (IQR, 3–7), and the median number of nephrology visits within 1 year after education session was 7 (IQR, 3–10).

#### Modality Choice and Initiation

**Figure 2:**
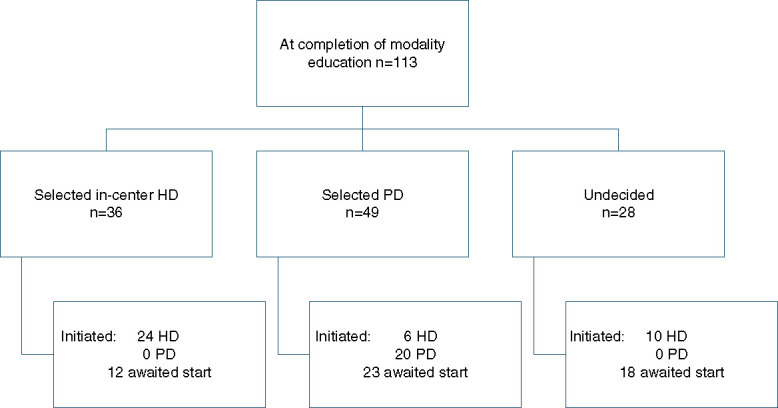
**Breakdown of dialysis decision-making after modality education and ultimate modality initiated.** PD, peritoneal dialysis.

At completion of the modality education, 28 (25%) selected in-center hemodialysis, 66 (58%) selected PD, and 19 (17%) were undecided. Hemodialysis was ultimately selected by 36 (32%), while 49 (43%) ultimately selected PD and 28 (25%) were ultimately undecided (Table 1). Of the 62 (56%) patients who started on any modality, two (3%) received a transplant, 20 (31%) initiated PD, and 40 (64%) initiated hemodialysis. Of the 49 who had ultimately selected PD, 20 (41%) initiated PD, 6 (12%) initiated in-center hemodialysis, and the remaining 23 had not initiated dialysis at the end of study period. Of the 28 patients who were ultimately undecided, 10 (36%) initiated in-center hemodialysis and zero initiated PD, while the remaining 18 awaited dialysis initiation. A total of 36 (32%) of the entire cohort required urgent hemodialysis initiation, including eight (16%) of those who had ultimately selected PD and 19 (53%) of those who ultimately selected hemodialysis (Figure 2).

#### Correlates of PD Choice and Initiation

Individuals who ultimately selected PD after education session were younger (median age 63 years [IQR, 51–68]) than those who selected hemodialysis (median age 66.5 years [IQR, 54–75]) and those who remained undecided (median age 68.5 years [59–75.5]; Table 1). Racial/ethnic characteristics did not differ between those who selected different dialysis modalities, but a higher proportion of individuals who identified as Hispanic ultimately selected hemodialysis rather than PD (45% versus 39%, respectively). Employment status, marital status, insurance status, and comorbidity score did not differ significantly between the groups, but those who ultimately selected PD had the lowest burden of comorbidities. Surprisingly, those who ultimately selected PD were referred to nephrology at a lower eGFR compared with those who did not (median eGFR 19 ml/min per 1.73 m^2^ [IQR, 9–26] versus 23.5 ml/min per 1.73 m^2^ [IQR, 16–31]). There was no difference between groups in eGFR at the time of modality education referral nor was there a difference in the duration between referral and completion of education. Interestingly, those individuals who ultimately selected PD had fewer attempts made by the education team to schedule education than those who were undecided or selected hemodialysis (median number of attempts: 1 [IQR, 1–3] versus 3 [IQR, 2–4.5] versus 2 [IQR, 1–5]), respectively (Table 1).

Compared with those individuals who selected PD and initiated PD, those individuals who initiated hemodialysis urgently were older (median age 54 years [IQR, 40–67] versus 66 years [IQR, 55–70], respectively), less likely to be employed (42% versus 12%), more likely to have diabetes, and less likely to have GN or polycystic kidney disease as their cause of kidney failure (21% versus 63%, and 47% versus 20%, respectively; Table 2). Furthermore, PD initiators had a lower burden of comorbidities and had a lower average eGFR at nephrology referral. The number of attempts made by educators to schedule modality education was fewer in PD initiators, as was the number of missed nephrologist appointments within 1 year before education, as compared with urgent hemodialysis initiators (Table 2).

**Table 2:** Comparison of those who ultimately selected PD and initiated PD compared with those urgently required hemodialysis regardless of ultimate modality choice

Variable	Elected PD and Started PD (*n*=19/113)	Required Urgent Hemodialysis (*n*=35/113)	*P* Value
Age	54 (40–67)	66 (55–70)	0.03
**Sex, *n* (%)**			0.4
Female	8 (42.1)	19 (54.3)	
**Race, *n* (%)**			0.8
Asian	0	1 (2.9)	
Black	8 (42.1)	15 (42.9)	
Other	7 (36.8)	15 (42.9)	
White	4 (21.0)	4 (11.4)	
**Ethnicity, *n* (%)**			0.6
Hispanic	11 (58.0)	18 (51.4)	
Married or significant other, *n* (%)	8 (42.1)	15 (42.9)	0.9
**Insurance, *n* (%)**			0.5
Yes	19 (100)	34 (97.1)	
**Employment status, *n* (%)**			0.08
Unemployed	4 (21.0)	10 (29.4)	
Employed	8 (42.1)	4 (11.8)	
Retired/disability	7 (36.8)	19 (54.2)	
Other	0	1 (2.9)	
**Cause of ESKD, *n* (%)**			0.01
DM	4 (21.0)	22 (62.9)	
HTN	6 (31.6)	4 (11.4)	
GN or PCKD	9 (47.4)	7 (20.0)	
Other	0	2 (5.7)	
**Charlson comorbidity score category, *n* (%)**			0.002
Low (2–5)	15 (79.0)	10 (29.0)	
Medium (6–7)	2 (10.5)	15 (42.9)	
High (8–15)	2 (10.5)	10 (28.6)	
eGFR at initial nephrology visit, median (IQR)	14 (7–24)	20 (14–29)	0.1
eGFR at modality referral, median (IQR)	10 (7–13)	11 (10–13)	0.1
Median number of days between referral and education, *N* (IQR)	22 (7–29)	21 (6–49)	0.6
No. of attempts made to schedule education, *N* (IQR)	1 (1–3)	3 (1–6)	0.05
Median number of nephrology appts scheduled in year before referral, *N* (IQR)	5 (1–7)	5 (3–6)	0.2
Median number of missed nephrology appt in the year before referral, *N* (IQR)	0 (0–2)	1 (0–3)	0.05
Nephrologist educated patient before referral, yes	16 (84.2)	28 (80.0)	0.7
Median number of contacts made by KS team 1 yr after education, *N* (IQR)	4 (2–7)	6 (3–7)	0.4

DM, diabetes mellitus; HTN, hypertension; IQR, interquartile range; PCKD, polycystic kidney disease; PD, peritoneal dialysis.

### Semistructured Interviews of Patients and Their Experience with Predialysis Education

#### Patient Characteristics

Of 37 participants approached, 13 were interviewed. Reasons for nonparticipation included hospitalization, lack of English comprehension, lack of technological comprehension, and difficulty scheduling. Six (46%) were female, the mean age was 63.5 years (Supplemental Table 1), and average length of interview was 32 minutes. Of the 13 interviewed, five had not started dialysis, three were receiving PD and five were receiving hemodialysis. Dialysis vintage was <1 year in those who were receiving PD or hemodialysis.

#### Themes

**Figure 3:**
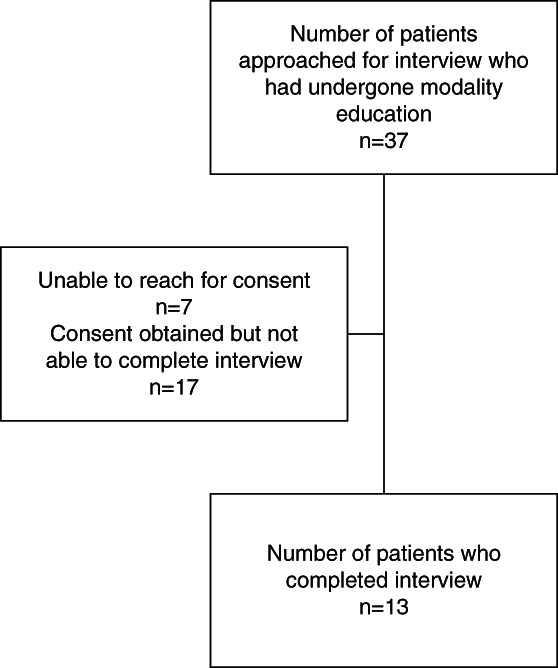
Consort diagram for interviewees.

**Figure 4:**
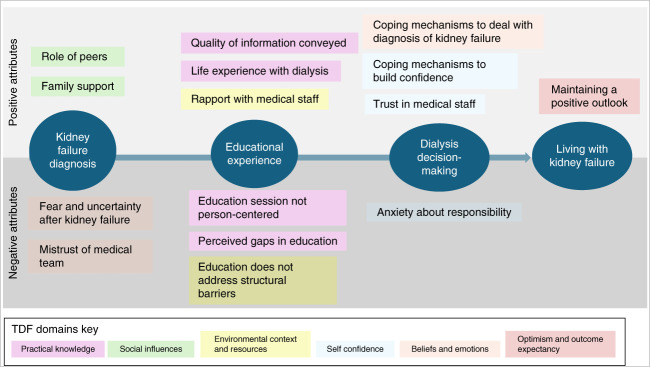
**Thematic schema illustrating TDF domains under trajectory of kidney failure timeline.** TDF, theoretical domain framework.

Qualitative analysis confirmed *a priori* themes grouped by TDF domain (Table 3). A thematic schema was created aligning these domains with the trajectory of dialysis decision-making (Figure 3). Participant perceptions of the trajectory of modality education session and their decision-making experience encompassed positive and negative attributes and represented occasional contradictory views within themes. Below, we present the findings in order of the trajectory of dialysis decision-making, listing the domains with associated themes in parentheses. Two TDF domains were associated with understanding decision-making around kidney failure: *social influences* (role of peers, family support) and *beliefs and emotions* (fear and uncertainty and mistrust of medical team). Most participants did not rely on family support to help them with decision-making, and there was a mix of feelings regarding the helpfulness of a peer, but most believed the insight offered from a fellow patient potentially helpful. Two TDF domains were associated with the experience of receiving education: *practical knowledge* (quality of information conveyed, life experience with dialysis, education session not person-centered, perceived gaps in education) and *environmental context* (rapport with medical staff, education does not address structural barriers). Overall, participants felt that the education session was not personalized to help them make informed decisions, and while it imparted knowledge and the staff providing education were supportive and comprehensive in their overview, most patients left feeling overwhelmed. Personal experiences with dialysis through witnessing loved ones helped some to gain a better understanding of practical aspects of PD. The education session did not seem to address structural barriers to home dialysis uptake such as the appropriate space, type of home environment, and how to navigate difficulty with finances. Rapport with staff was generally good, but there was an impression that the staff did not try hard enough to understand the challenges imposed by structural barriers and instead followed a depersonalized protocol which was formal and not sensitive to their lived experiences. Some expressed mistrust of the intentions of the providers and believed they were financially motivated (Figure 4).

**Table 3:** Results of qualitative analyses of 13 interviews: domains and themes with representative quotes from interviewees

**TDF domain: modality education session imparting practical knowledge**	
Definition: knowledge of kidney disease and practical aspects of the modality	
Theme: education session not person-centered	“It kind of felt like there was a mold. There was a certain archetype that I had to follow, and if I didn't follow this, it wasn't going to work out. So, *i.e*., another thing that pushed me toward the fistula in the arm” (D) “I resented the way it was being presented because it was a format and they have to follow this protocol where life is not like that.” (J) “I felt that they did not know what I was going through…their job is exactly that, a job they studied for…they don't get it because you're not wearing these shoes” (J)
Theme: quality of information conveyed was generally good with good support from staff	“The staff was excellent, we talked about a lot of different stuff, leading up to dialysis. She was informative and pleasant. She gave me a lot of information if I wanted to have it at home” (C) “She brought over dummies, it was very informative, she gave me literature to read” (D) “It was a nurse, who was teaching “They were very patient and … they took time and they brought my daughter in the call and they made [her feel] comfortable too” (K)
Theme: life experience with dialysis	“I was familiar with it [medical terminology] because I'm a CNA. So I was kind of sort of familiar with the stuff, you know, like dealing with patients that had to do dialysis and stuff like that” (A) “My sister was on hemodialysis and she was very resistant and she wind up passing away. Well, three times she got on the dialysis machine and….so they had to revive her the last time they didn't revive her” (C) “I also had an older sister…she did hemo and I remember I was with her for a lot of it… It was just awful to watch” (J)
Theme: perceived gaps in educational program-answers to practical questions	“I was thinking about how if you were sleeping by yourself in the room and then you have to do it so if you want to go to the bathroom…I was thinking about how to get the materials to do it” (A) “There was some drawbacks that were kind of hard to figure out- the storage of the fluid and how did you get rid of the fluid when you're finished …I didn't realize there were 20 bags of fluid involved and again how do you dispose of it when you're finished” (G) “I'm not quite sure if I'm kind of trying to figure out how big is the machine. That was never made clear to me. They showed me the blood machine, the portable that at home dialysis machine. But the, I assume that the one with the water is bigger, but I don't know if that's true or not”(D)
**TDF domain: social influences**	
Definition: how family, friends or peers can support modality decision-making	
Theme: role of peers in dialysis decision-making and transition	“I'm going through within the confines of my life even though it's nice to hear one of the people going through which I do go online and I see Youtube videos or people's testimonial” (D) “I think it would be a little too heartbreaking…to start hearing people that are going through it…I don't think I would have focused on anything except that their story is…and I would have gone into emotional about it” (D) “I feel like [peers] could help you know to show that you know you're not, you're not alone in this…to show moral support if at least anything else, especially for somebody who may not have somebody with them” (I) “They [paired] me up for training with a gentleman. …we ended up being supportive for each other” (J)
Theme: family support with dialysis decision-making	“I don't have much support. My wife only” (B) “I'm my support system…I made my decision on my own” (C) “I have people that love me. I have people that care, but I also realize that people are too busy. So this is the hand that I was delt with so I have to live with it” (J)
**TDF domain: environmental context and resources**	
Definition: having the appropriate space, location, and informational resources to start dialysis	
Theme: education does not address practical aspects of future on dialysis	“I understand that doctors are trying to help and keep you alive, but rent is not free. And to me, *i.e*., the number one thing I think of when I am stuck in a hospital. I live paycheck to paycheck, and every day that I am here, I'm getting paid less. So it is a matter of not putting money and an apartment above my life, but it has to be worked in, to be negotiated around it” (D) “Education session did not talk about work or finance, and sometimes finance is a big deal when you are on dialysis. I know how much money I have spent out of pocket myself since ’95. I know how expensive it is. They should discuss the finances, and if you are employed, and what can they do to keep their jobs and navigating employers with the schedules of dialysis and how often you come in. For the ones who want to work” (C) “I live in a very small apartment, I am single, my family lives very far away from me. So immediately I thought this was not going to work out for me at home” (D)
Theme: rapport with staff	“It was when I met [the doctor] that she allayed my fears and she gave me hope” (J) “The people they didn't mind me asking for repetition, they were very patient with me. They made sure I was comfortable. They involved my daughter in the call and made her feel comfortable too” (K) “The education session was a little scary because it was new territory for me to understand and learn the seriousness of this. Not to take it so lightly. It did scare me a little, but I feel comfortable with it. They were very supportive, very supportive, very informative of my option … what does it mean for me long term and short term of living in life” (I)
**TDF domain: self confidence**	
Definition: Having enough confidence to handle their diagnosis and treatment	
Theme: anxiety about responsibility (emotional trauma)	“I don't mind if I have to do this dialysis in my home. I would just sit back and watch some TV. As horrible as dialysis is, if I could just be relaxed, it's not a big deal. But once they told me the information about all the equipment, and the supplies and the constant deliveries, and that I might need someone's help” (D) “It was a lot of decision making that I had to do moving forward. Even getting the surgery was terrifying to do. My partner was the one who really helped me make the decisions now it sounds complicated” (E) “Peritoneal is definitely a better choice but not for me at the moment…I'm a little too fresh and I'm nervous… everything has to be sterile…I don't want to mess up anything and find myself in a bad situation” (D)
Theme: trust in medical staff	“I believe everything was ok. They made sure that I left there not crying anymore and feeling like it will be ok. To take my time and be accurate with which one I want to do, because I am going to have to start soon. “(E) “I did feel like they were sympathetic to me having to go through this for the first time. That I was being resistant, but I have to do it. They were not getting together, they said to take your time. Digest everything you are telling me” (E) “It made feel empowered. The education was given very directly and I was able to understand what it is I had to choose from and able to make a decision” (I)
Theme: coping mechanisms to build confidence	“I do feel like I am making a big step and I am taking my health into consideration more so than working or anyone else. I am taking charge of me, I am choosing me for a change” (E) “When it is time for you to have it, you have to be brave and go through with it” (F) “I'm a little scared about it, but if you know if it's gonna prolong my life, then I'm just gonna have to do what I have to do” (K)
**TDF domain: beliefs and emotions**	
Definition: the perceptions patients have about their diagnosis, disease, and treatment options and the way in which they handle these feelings	
Theme: fear and uncertainty after diagnosis of kidney failure	“I was afraid…I knew right away that my life has changed” [A] “It impacted the quality of my life and the trajectory of my life. It affected my life by more or less being vigilant, almost to paranoia about what I was consuming. Trying to get as much information as I could” (C) “I was sad for sure because there was nothing I could do about it…it was taken out of my hands” (J) “It felt like the rug was pulled right from under me” [J] “Automatically I thought I was going to die. My uncle had to do dialysis and 12 mo after, he passed away. That was my biggest fear” [K]
Theme: medical mistrust	“Why should I pay another fare for them…why don't they just do it free…they want to take money off of you again like always” (A) “I used to go to [somewhere] every year, with dialysis I can't. Why can't they do it free? Why do they have to charge. You're already going through a bad experience and they want to make more money. Everything is money. Everything changes” [B]
Theme: coping mechanisms to deal with diagnosis of kidney failure	“I feel I do not have a choice. If I had a choice, I would not go through dialysis or learning about it…but my lifespan is going to change” (B) “I'm just so angry all the time. It's up to the situation and it's not important enough to anybody else except me” [J] “I've started rewatching a lot of old TV and a comedian…to give me a few laughs…coping in that way” [D] “Leave it to God. I cannot do anything about it” [M] “Education session did not incorporate faith enough. There are a lot of African Americans who go through renal failure” (C)
**TDF domain: optimism and outcome expectancy**	
Definition: The perceptions people have regarding their future living with the new diagnosis and dialysis options and the results they expect	
Theme: coping mechanisms for living life with kidney disease	“I believe I am blessed and lucky, because with everything going on with me, I should have been gone a long time ago. The way I was raised and my family, I never give up. I try to fight through everything. I keep my appointments, I do it for my family. I pray, that helped me through a lot of times too. My upbringing, my family, my religion. God helps those who help themselves” [C] “I was raised in the church, that did not affect me. I believe that God allows me to make a decision that I will be open to it. I was open to it by going to the class and getting educated on it” (K) “There are people who will tell you your life is not going to be the same. Some people are on dialysis for a long time. Some say dialysis will save life, you don't have any other way to go than to do dialysis. That is the only way to preserve your life. You reach a point where you don't have any other way of going” [F] “My shortness of breath might get better, my cramping will get a little better. The itching will get better. That was definitely reassuring” [E] “I am still in a state of panic, I am still scared, regardless of me taking care of myself, doing the right thing, and knowing that this will give me a longer life until I get a kidney” [L]

Please see Supplemental Table 1 for letter designations of the interviewees. CNA, certified nursing assistant; TDF, theoretical domain framework; TV, television.

Two TDF domains were associated with inner drivers of dialysis decision-making: *self-confidence* (coping mechanisms to build confidence, trust in medical staff, anxiety about responsibility) and *beliefs and emotions* (coping mechanisms to deal with diagnosis of kidney failure). Most participants lacked self-confidence regarding their abilities and were apprehensive regarding taking on the responsibility of treatment and maintaining their own safety. Most felt that the staff providing education were patient with them and tried to build up their confidence. Participants described the immense emotional trauma, described as fear, uncertainty, and sense of doom surrounding their diagnosis, as a barrier to decision-making and comprehension of concepts presented to them during the education. They felt alone in their struggles to cope with the large emotional toll this decision-making took on them. Most described resignation to the inevitable and channeling faith and distractions as their coping mechanisms. Most were resolute about survival and believed the dialysis staff would support them. One TDF domain, *optimism and outcome expectancy*, addressed outlook while living with kidney failure. Participants described maintaining a positive outlook on their kidney disease through faith and resolve, which made them feel less overwhelmed regarding decision-making.

## Discussion

This study evaluated correlates of PD uptake and perceptions of a standardized educational program in a cohort of minoritized populations, a group disproportionately affected by kidney failure, who has been understudied. Because dialysis modality education has been positively associated with PD uptake, understanding the shortcomings of current educational models is critical to personalize and improve these sessions. Among patients who attended modality education, those with fewer comorbidities and who were easier to schedule for education ultimately selected PD and were initiated on PD more often than older individuals, those with more comorbidities, and those who were more difficult to engage by scheduling teams and nephrologists. We measured patient responsiveness to attempts made by staff by number of contact attempts made and number of nephrology appointments attended after education session. Surprisingly, individuals who ultimately selected and were initiated on PD were referred to nephrology at a significantly lower eGFR than individuals who ultimately selected hemodialysis or were urgently started on hemodialysis. The reason for this finding may be that because PD initiators were healthier, the primary care physicians did not feel a need for guidance from a nephrologist until later in their disease course. Thus, it may take a deliberate effort and persistence of attempts to engage with patients with higher burden of disease and higher risk of rapid CKD progression.

Our qualitative analysis revealed that while patients felt overwhelmed by the information presented in modality education and felt as if the sessions were not personalized to them or their practical needs and did not address the emotional trauma of their diagnosis, they were receptive to support and guidance provided by education staff and peers. Previous studies have highlighted the importance of the modality education experience in selecting home dialysis modalities. Among 427 patients receiving dialysis in the United States, the probability of selecting PD was independently associated with PD being presented as a dialysis option and an increased length of time spent discussing treatment options.^16^ Dialysis choice came as a shock for half of the patients in one study, and the magnitude of the decision to initiate dialysis seemed rushed and overwhelming, even in those who had been seeing a nephrologist for years.^17,18^ Elements of the modality education that acknowledge patients experiences and autonomy increased patient engagement, while threats to participant autonomy through disregarding their narrative or providing education discordant to their specific psychosocial needs exacerbated mistrust and perception of threat. In one study, over half of the patients felt alone when asked to decide about dialysis initiation, and some felt that the bad news was delivered without much sensitivity.^19^ The uncertainty and disruption of self-identity were compounded when patients' emotional and psychosocial needs were overlooked.^6^ Participants described pushing the limits of their health and fighting for autonomy while navigating health care systems that disregarded their self-reported social, emotional, or physical needs.

Standardizing a method to personalize modality education is an important goal. We were unable to demonstrate the effectiveness of repeating modality education on decision-making, but showed that most patients felt that integration of faith and coping mechanisms would be beneficial to their decision-making and may ameliorate feelings of anxiety. Training educators on emotional trauma, motivational trauma and giving space during the education for reflection may further help with navigating the emotional side of a new diagnosis of kidney failure, including acknowledging and normalizing the emotional trauma, establishing rapport and trust, and setting expectations for the modality education session. Furthermore, understanding how different populations cope with chronic disease, such as incorporating faith and practical coping mechanisms and understanding health literacy level of the patient is critical, and educators should strive to meet the patient population where they are in their journey.

Ours is a single-center study, albeit one with a large proportion of minoritized individuals, and although the cohort was prospectively collected, the analysis was retrospective. We used a convenience sample of patients, thus they do not represent patients who are not seen by nephrology or were seen too late in their disease course to allow for modality education. Qualitative interviews may not have probed certain aspects of shared decision-making. Furthermore, the interview transcripts did not ask specifically about why some patients changed their minds about which modality they initiated, after initially selecting a different modality, nor was there an evaluation of knowledge uptake from the education session as a driver of modality selection.There was risk of recall bias in those whose interviews took place sometime after the modality education and especially in those who had already initiated dialysis. Finally, some patients in the early 2021 period may have faced barriers to engaging in modality education brought about by coronavirus disease restrictions.

We found gaps in tailoring modality education to the needs of minoritized individuals which involve considerations along the trajectory of kidney failure timeline. Early referral with plenty of time for shared decision-making and coping with emotional trauma, leveraging peer and family support for dealing with kidney failure as a diagnosis, person-centered education, and consideration of structural barriers and life experience while supporting a positive outlook with respect to living with kidney failure are all consideration for future design of modality education.

## Supplementary Material

SUPPLEMENTARY MATERIAL

## Data Availability

Partial restrictions to the data and/or materials apply. Will get approval from IRB and National Kidney Foundation for release of this data.

## Supplemental Material

Supplemental Table 1. Characteristics of interviewees.

PD Modality Education and Barriers Project
