# Supplementary material for: Uptake of Peritoneal Dialysis by Minoritized Patients: The Role of Modality Education
Source: Kidney360. 2025 Feb 3;6(5):793–804. doi: 10.34067/KID.0000000702 (PMC12136653; doi:10.34067/KID.0000000702)
Supplement: SUPPLEMENTARY MATERIAL [file kidney360-6-793-s001.pdf]

ASN American Society of Nephrology JASN Journal of the American Society of Nephrology KIDNEY360 Accessing Our World From Every Angle CJASN Clinical Journal of the American Society of Nephrology

ASN Journal Disclosure Form

As per ASN journal policy, I have disclosed any financial relationships or commitments I have held in the past 36 months as included below. I have listed my Current Employer below to indicate there is a relationship requiring disclosure. If no relationship exists, my Current Employer is not listed.

I. Arnaoudova reports the following:
Employer: Albert Einstein College of Medicine; Meta; and Ownership Interest: Delta Airlines.

I understand that the information above will be published within the journal article, if accepted, and that failure to comply and/or to accurately and completely report the potential financial conflicts of interest could lead to the following: 1) Prior to publication, article rejection, or 2) Post-publication, sanctions ranging from, but not limited to, issuing a correction, reporting the inaccurate information to the authors' institution, banning authors from submitting work to ASN journals for varying lengths of time, and/or retraction of the published work.

Name: Ivelina Arnaoudova
Manuscript ID: K360-2024-000895R1
Manuscript Title: Uptake of Peritoneal Dialysis by Minoritized Patients-The Role of Modality Education
Date of Completion: December 2, 2024
Disclosure Updated Date: December 2, 2024

ASN American Society of Nephrology
JASN Journal of the American Society of Nephrology
KIDNEY360 Accessing Our World From Every Angle
CJASN Clinical Journal of the American Society of Nephrology

ASN Journal Disclosure Form

As per ASN journal policy, I have disclosed any financial relationships or commitments I have held in the past 36 months as included below. I have listed my Current Employer below to indicate there is a relationship requiring disclosure. If no relationship exists, my Current Employer is not listed.

N. Ehtesham reports the following:
Employer: Albert Einstein College of Medicine

I understand that the information above will be published within the journal article, if accepted, and that failure to comply and/or to accurately and completely report the potential financial conflicts of interest could lead to the following: 1) Prior to publication, article rejection, or 2) Post-publication, sanctions ranging from, but not limited to, issuing a correction, reporting the inaccurate information to the authors' institution, banning authors from submitting work to ASN journals for varying lengths of time, and/or retraction of the published work.

Name: Nahian Ehtesham
Manuscript ID: K360-2024-000895R1
Manuscript Title: Uptake of Peritoneal Dialysis by Minoritized Patients-The Role of Modality Education
Date of Completion: December 5, 2024
Disclosure Updated Date: December 5, 2024

ASN American Society of Nephrology | JASN Journal of the American Society of Nephrology | KIDNEY360 Accessing Our World From Every Angle | CJASN Clinical Journal of the American Society of Nephrology

ASN Journal Disclosure Form

As per ASN journal policy, I have disclosed any financial relationships or commitments I have held in the past 36 months as included below. I have listed my Current Employer below to indicate there is a relationship requiring disclosure. If no relationship exists, my Current Employer is not listed.

L. Golestaneh reports the following:
Employer: Yale University School of Medicine; Consultancy: Horizon Pharmaceuticals and Amgen rare diseases; Research Funding: Atra Zeneca; Honoraria: Horizon Pharmaceuticals: $4000;; WebMD $2500; Advisory or Leadership Role: Advances in Chronic Kidney Disease; Speakers Bureau: National Kidney Foundation Talk sponsored by CoreMedix; and Other Interests or Relationships: I receive compensation from the Cardiovascular Research Foundation for fulfillment of duties as a member of the Clinical Events Committee for the the Spyral Hypertension trials, sponsored by Medtronic; I serve on the CEC committee for Axon Therapeutics; Part of my salary is paid for by Yale's CORE.

I understand that the information above will be published within the journal article, if accepted, and that failure to comply and/or to accurately and completely report the potential financial conflicts of interest could lead to the following: 1) Prior to publication, article rejection, or 2) Post-publication, sanctions ranging from, but not limited to, issuing a correction, reporting the inaccurate information to the authors' institution, banning authors from submitting work to ASN journals for varying lengths of time, and/or retraction of the published work.

Name: Ladan Golestaneh
Manuscript ID: K360-2024-000895R1
Manuscript Title: Uptake of Peritoneal Dialysis by Minoritized Patients-The Role of Modality Education
Date of Completion: November 19, 2024
Disclosure Updated Date: October 12, 2024

ASN American Society of Nephrology
JASN Journal of the American Society of Nephrology
KIDNEY360 Accessing Our World From Every Angle
CJASN Clinical Journal of the American Society of Nephrology

ASN Journal Disclosure Form

As per ASN journal policy, I have disclosed any financial relationships or commitments I have held in the past 36 months as included below. I have listed my Current Employer below to indicate there is a relationship requiring disclosure. If no relationship exists, my Current Employer is not listed.

K. Rizzolo has nothing to disclose.

I understand that the information above will be published within the journal article, if accepted, and that failure to comply and/or to accurately and completely report the potential financial conflicts of interest could lead to the following: 1) Prior to publication, article rejection, or 2) Post-publication, sanctions ranging from, but not limited to, issuing a correction, reporting the inaccurate information to the authors' institution, banning authors from submitting work to ASN journals for varying lengths of time, and/or retraction of the published work.

Name: Katherine M. Rizzolo
Manuscript ID: K360-2024-000895R1
Manuscript Title: Uptake of Peritoneal Dialysis by Minoritized Patients-The Role of Modality Education
Date of Completion: November 19, 2024
Disclosure Updated Date: November 19, 2024

ASN American Society of Nephrology
JASN Journal of the American Society of Nephrology
KIDNEY360 Accessing Our World From Every Angle
CJASN Clinical Journal of the American Society of Nephrology

ASN Journal Disclosure Form

As per ASN journal policy, I have disclosed any financial relationships or commitments I have held in the past 36 months as included below. I have listed my Current Employer below to indicate there is a relationship requiring disclosure. If no relationship exists, my Current Employer is not listed.

J. Shen reports the following:
Employer: Lundquist Institute at Harbor-UCLA Medical Center; Consultancy: Outset Medical; Healthmap; and Other Interests or Relationships: National Kidney Foundation; American Society of Nephrology;; International Society of Peritoneal Dialysis; PDOPPS.

I understand that the information above will be published within the journal article, if accepted, and that failure to comply and/or to accurately and completely report the potential financial conflicts of interest could lead to the following: 1) Prior to publication, article rejection, or 2) Post-publication, sanctions ranging from, but not limited to, issuing a correction, reporting the inaccurate information to the authors' institution, banning authors from submitting work to ASN journals for varying lengths of time, and/or retraction of the published work.

Name: Jenny I. Shen
Manuscript ID: K360-2024-000895R1
Manuscript Title: "Uptake of Peritoneal Dialysis by Minoritized Patients-The Role of Modality Education
Date of Completion: November 18, 2024
Disclosure Updated Date: October 29, 2024

ASN American Society of Nephrology | JASN Journal of the American Society of Nephrology | KIDNEY360 Accessing Our World From Every Angle | CJASN Clinical Journal of the American Society of Nephrology

ASN Journal Disclosure Form

As per ASN journal policy, I have disclosed any financial relationships or commitments I have held in the past 36 months as included below. I have listed my Current Employer below to indicate there is a relationship requiring disclosure. If no relationship exists, my Current Employer is not listed.

C. Wilson reports the following:
Employer: Hospital of University of Pennsylvania

I understand that the information above will be published within the journal article, if accepted, and that failure to comply and/or to accurately and completely report the potential financial conflicts of interest could lead to the following: 1) Prior to publication, article rejection, or 2) Post-publication, sanctions ranging from, but not limited to, issuing a correction, reporting the inaccurate information to the authors' institution, banning authors from submitting work to ASN journals for varying lengths of time, and/or retraction of the published work.

Name: Clara Wilson
Manuscript ID: K360-2024-000895R1
Manuscript Title: Uptake of Peritoneal Dialysis by Minoritized Patients-The Role of Modality Education
Date of Completion: November 19, 2024
Disclosure Updated Date: November 19, 2024

ASN American Society of Nephrology
JASN Journal of the American Society of Nephrology
KIDNEY360 Accessing Our World From Every Angle
CJASN Clinical Journal of the American Society of Nephrology

ASN Journal Disclosure Form

As per ASN journal policy, I have disclosed any financial relationships or commitments I have held in the past 36 months as included below. I have listed my Current Employer below to indicate there is a relationship requiring disclosure. If no relationship exists, my Current Employer is not listed.

J. Wilson reports the following:
Employer: DaVita Kidney Care

I understand that the information above will be published within the journal article, if accepted, and that failure to comply and/or to accurately and completely report the potential financial conflicts of interest could lead to the following: 1) Prior to publication, article rejection, or 2) Post-publication, sanctions ranging from, but not limited to, issuing a correction, reporting the inaccurate information to the authors' institution, banning authors from submitting work to ASN journals for varying lengths of time, and/or retraction of the published work.

Name: Jennie Wilson
Manuscript ID: K360-2024-000895R1
Manuscript Title: Uptake of Peritoneal Dialysis by Minoritized Patients-The Role of Modality Education
Date of Completion: January 9, 2025
Disclosure Updated Date: January 9, 2025
