# Supplementary material for: Uptake of Peritoneal Dialysis by Minoritized Patients: The Role of Modality Education
Source: Kidney360. 2025 Feb 3;6(5):793–804. doi: 10.34067/KID.0000000702 (PMC12136653; doi:10.34067/KID.0000000702)
Supplement: SUPPLEMENTARY MATERIAL [file kidney360-6-793-s002.pdf]

Supplemental Index:

Supplemental Table 1: Characteristics of Interviewees

**Subject ID**	**Age**	**Race**	**Ethnicity**	**Sex**	**Dialysis Modality**
A	67	Black or African-American	Not Spanish/Hispanic/Latino	Female	In-Center hemodialysis
B	72	Other	Spanish/Latin o/Hispanic	Male	Not on dialysis, CKD Stage 5
C	70	Black or African-American	Not Spanish/Hispanic/Latino	Female	Not on dialysis, CKD Stage 5
D	54	Black or African-American	Not Spanish/Hispanic/Latino	Female	In-Center hemodialysis
E	80	Black or African-American	Not Spanish/Hispanic/Latino	Male	In-Center hemodialysis
F	38	Other	Spanish/Latino/Hispanic	Male	In-Center hemodialysis
G	48	Black or African-American	Not Spanish/Hispanic/Latino	Male	Peritoneal Dialysis
H	84	Black or African-American	Not Spanish/Hispanic/Latino	Female	Not on dialysis; CKD stage 5
I	68	Other Race	Spanish/Latino/Hispanic	Female	Peritoneal dialysis
J	45	Black or African-American	Not Spanish/Hispanic/Latino	Female	Not on dialysis; CKD Stage 5
K	55	Declined race	Spanish/Latino/Hispanic	Male	Peritoneal dialysis
L	67	Black or African-American	Not Spanish/Hispanic/Latino	Male	Hemodialysis
M	78	Black or African-American	Not Spanish/Hispanic/Latino	Male	Not on dialysis; CKD Stage 5

<u>Peritoneal Dialysis Modality Education and Barriers Project</u>

Interview Guide

- Thank you for meeting to discuss your experience with the Dialysis Modality Education. This interview will be about 30 minutes long and will aim to understand some barriers or reservations you may feel when considering peritoneal dialysis as your modality of choice. We would also like to know if/how you think the educators understood your particular-situation and what influenced your decision making.
- We need your honest feedback to identify the parts of the dialysis modality education that were easiest to understand and what needs to be improved to help patients make good decisions.
- At the end of our discussion, I will give you the details for an Amazon gift card as a thank you for participating.
- Before we start, is it ok if I record this interview?
- I will first start by asking a few questions.
  - Can you please tell me who referred you for dialysis modality education? And when?
  - Who is a part of your support system in making this decision?
  - How far did you go in school?

Let's start with your assessment of the dialysis education overall.

Interview question	Conceptual construct	CFIR resource
1. First, what were your biggest concerns when you were first diagnosed with chronic kidney disease?<br>a. How did this diagnosis impact your day to day life?<br>b. How do you feel that your nephrologist, dialysis education team, and other healthcare providers provided emotional support during this time? What would you have seen done differently?<br>c. Did you have any hesitations to reach out the providers or staff about questions or to share your concerns?<br>d. When you were first told that you may require dialysis in the future, how did you feel and how did you cope with this news?<br>e. What are your fears surrounding end stage renal disease and dialysis?<br>f. How confident do you feel in making the decision about dialysis?	introduction to better understand the emotional trauma of getting the diagnosis and coping mechanisms.<br><br>Acceptability	*You will have to be listening with a "third ear" for the constructs in CFIR and probe to be sure you can code them. Maybe have a list of the codes you might use and be sure the respondent's answer is clear enough that you can code it. For example: barriers, facilitators, mediators, individual beliefs, etc.*

2. What do you think of the dialysis education session that you had?<br><br>*(Probe):*<br>• *What did you like? Not like? Ask for examples.*		
3. Did you feel you had enough time before starting dialysis to attend the education session and think about your decision?<br>a. What were some of the delays, if any? Why did you experience some of these delays?<br><br>b. Were you able to review the reading materials?<br><br>c. Were you able to make a non-rushed decision?<br><br>d. Would you have preferred more than one modality education session?<br><br>e. Do you prefer an in-person session or a virtual session?<br><br>*(Probe):*<br>• *Why or why not?*<br>• *When/where would you ideally have liked to receive this dialysis modality education?*	Effectiveness<br><br>Appropriateness	CFIR Characteristics of Individuals: Knowledge and Beliefs about the Intervention<br>CFIR Intervention Characteristics: Compatibility
4. Was the information easy to understand?<br><br>a. Who led the class? RN, PCT, MD, Other?<br>b. Was there a problem understanding the material presented?<br><br>*(probe):*<br>• *Was there enough information? Too much?*<br><br>c. How did you feel about the medical vocabulary used and the steps in training/treatment for peritoneal dialysis?<br><br>d. Did you feel comfortable asking the staff any questions about dialysis?	Appropriateness<br><br>Feasibility, acceptability, effectiveness	CFIR Inner Setting: Patient Needs and Resources<br>CFIR Intervention Characteristics: Design Quality and Packaging

(probe):<br>• *Did the educator help you feel confident or did s/he scare you?*<br><br>e. What about the peritoneal dialysis education session did you find encouraging?<br><br>f. What part of the session did you find intimidating or discouraging?<br><br>*5.* After the dialysis modality education, how did it impact your confidence in dialysis and your decision for which modality to choose?<br><br>*(probe):*<br>• *Was the material presented in a way that was relevant to your day-to-day life?*<br>• *Was it easy to understand the different types of dialysis?*<br><br>*(Probe): after the respondent answers, probe if not mentioned:*<br><br>• *How was the education flow?*<br>• *How was the material presented to you (video, booklet, pamphlet, group conversations with other patients, patient decision aids, etc)?*<br>• *Anything you would like to change in the way the information was presented?*		CFIR Characteristics of Individuals: Self-efficacy

Now I am going to ask about some specific aspects of the education.

Interview question	Conceptual construct	CFIR resource
6. How did you feel that the dialysis education session incorporated your support system (family or friends that help you with your health decisions)?<br>a. Was there an understanding and proper consideration of your support system by the educator?<br><br>b. Would you prefer talking to other peers who are undergoing peritoneal dialysis as a part of the education process? How do you think this would impact your decision	Feasibility<br>Acceptability<br>Effectiveness	CFIR Intervention Characteristics: Compatibility<br>CFIR Intervention Characteristics: Complexity<br>CFIR Intervention Characteristics: Design Quality and Packaging

and sense of empowerment to choose this dialysis modality?<br><br>*(Probe):*<br><br>• *How would you change the education session to incorporate your support system more?*<br><br>• *Did other peers who are undergoing dialysis now influence your dialysis education process and decision?*<br>7. How did your faith or beliefs or your cultural habits affect the dialysis modality education process and decision for/or against peritoneal dialysis?<br><br>*(Probe):*<br>• *How do you feel that the dialysis modality education did in incorporating your faith or beliefs or your cultural habits? What do you feel should change to do so?*<br><br>8. How do your job and financial situation/living situation influence your dialysis modality education process and decision?<br><br>*(Probe):*<br>• *Do you feel that the dialysis modality education did in incorporating your job/financial situation, and living situation? What do you feel should change to do so?*		
9. At the end of the education session, how confident did you feel about deciding to choose peritoneal dialysis? On a scale of 1 to 10, 10 being the most confident.<br>a. How did you perceive your role in the decision-making process for the type of dialysis you chose at the end? Did you feel you had an active, collaborative, or passive role?<br>b. Looking back, do you feel the education session helped you feel empowered to make a decision about dialysis? How could this be done better?<br><br>c. Did you feel that you had adequate input into your decision for dialysis at the end of this process?	Acceptability	CFIR Intervention Characteristics: Design Quality and Packaging

d. What were some barriers to choosing peritoneal dialysis?<br><br>e. What about the education session was encouraging for you to choose peritoneal dialysis?<br><br>*(Probe):*<br>• *What factors went into your decision for peritoneal dialysis vs. hemodialysis?*<br><br>*If patient has trouble eliciting factors, can ask about specific factors: level of independence, quality of life, daily schedule, influence by family and peers on dialysis, confidence in doing peritoneal dialysis on own, space for supplies, etc.*		
10. What changes to the modality education do you think would encourage more patients like you to do PD?<br><br>*(Probe):*<br>• *If you could add or remove anything from the current dialysis education session, what would it be?*	Appropriateness	CFIR Intervention Characteristics: Design Quality and Packaging<br>CFIR Intervention Characteristics: Compatibility<br>CFIR Intervention Characteristics: Adaptability
